# Treatment of Cushing’s syndrome with osilodrostat: practical applications of recent studies with case examples

**DOI:** 10.1007/s11102-022-01268-2

**Published:** 2022-08-24

**Authors:** Maria Fleseriu, Beverly M. K. Biller

**Affiliations:** 1 Oregon Health & Science University, 3303 SW Bond Ave, Portland, OR 97239 USA; 2 Massachusetts General Hospital, Boston, MA USA

**Keywords:** Cushing’s syndrome, Cushing’s disease, Osilodrostat, Adrenal steroidogenesis inhibitors, Pituitary adenoma, Medical therapy

## Abstract

Endogenous Cushing’s syndrome (CS) is a rare endocrine condition frequently caused by a tumor resulting in elevated cortisol levels. Cushing’s disease (CD) caused by an adrenocorticotropic hormone–secreting pituitary adenoma is the most common form of endogenous CS. Medical therapy for CD is mostly used as second-line treatment after failed surgery or recurrence and comprises several pituitary-directed drugs, adrenal steroidogenesis inhibitors, and a glucocorticoid receptor blocker, some of which are US Food and Drug Administration (FDA)–approved for this condition. The recent Pituitary Society consensus guidelines for diagnosis and management of CD described osilodrostat, an oral inhibitor of 11β-hydroxylase, as an effective, FDA-approved medical therapy for CD. Because clinical experience outside clinical trials is limited, we provide here a review of published data about osilodrostat and offer example case studies demonstrating practical considerations on the use of this medication. Recommendations regarding osilodrostat are provided for the following situations: specific assessments needed before treatment initiation; monitoring for adrenal insufficiency, hypokalemia, and changes in QTc; the potential value of a slow up-titration in patients with mild disease; managing temporary treatment cessation for patients with CD who have acquired coronavirus disease 2019; monitoring for increased testosterone levels in women; exercising caution with concomitant medication use; considering whether a higher dose at nighttime might be beneficial; and managing cortisol excess in ectopic and adrenal CS. This review highlights key clinical situations that physicians may encounter when using osilodrostat and provides practical recommendations for optimal patient care when treating CS, with a focus on CD.

## Introduction

Endogenous Cushing’s syndrome (CS) is a serious and rare endocrine disorder resulting from chronic excess cortisol production [1, 2]. The presence of a pituitary adenoma that secretes adrenocorticotropic hormone (ACTH), known as Cushing’s disease (CD), is the most common cause of endogenous CS [1–3]. Optimal patient outcomes require management of the disease and the many associated comorbidities [1]. Myocardial infarction, stroke, and other vascular events account for the increased standard mortality ratio observed in patients with active CD [1]. The goals of CD treatment are to normalize the elevated cortisol levels, achieve tumor control with minimal hypopituitarism, manage comorbidities, and improve quality of life, with patient responses defined by both biochemical and clinical endpoints [1].

First-line treatment for most patients with CD is transsphenoidal surgery, with medical therapy used for persistent or recurrent hypercortisolism [1]. Pituitary-directed drugs, a glucocorticoid receptor blocker, and several adrenal steroidogenesis inhibitors (Table 1) can be used to treat CS/CD. It is important to note that study designs for medical therapies used to treat CS/CD are different, with no head-to-head comparisons available and, for some drugs, only retrospective data are available [1, 2, 4, 5]. Steroidogenesis inhibitors act on adrenal enzymes at multiple levels with varying selectivity and potency and provide the therapeutic benefit of a rapid onset of action (with the exception of mitotane) [2]. Osilodrostat is a potent, oral, reversible inhibitor of both 11β-hydroxylase, which catalyzes the final step of cortisol synthesis, and aldosterone synthase, which catalyzes conversion of 11-deoxycorticosterone to aldosterone [2]. Clinical evidence from the LCI699 in Cushing’s (LINC) studies have demonstrated the efficacy and safety of osilodrostat [6–9]. This medication is approved in the United States for the treatment of CD in adults for whom pituitary surgery is not an option or has not been curative [10], and it is approved for the treatment of all causes of endogenous CS in Europe and Japan.

**Table 1:** Summary of adrenal steroidogenesis inhibitors (adapted with permission from Fleseriu et al. [1])

Treatment	Frequency	Total daily dose: most frequently used (maximum)	Approval for CS or CD as of 2021^a^	Clinical considerations
*Oral treatments, alphabetical order*				
Ketoconazole [4, 24, 62]	BID or TID	400–1600 mg/day (1600 mg/day)	US (off-label use) EU (EMA for CS)	Risk of serious hepatotoxicity; monitor liver enzymes before and during treatment
				Risk of QTc prolongation
				Avoid use of gastric acid suppressors (e.g., H2-receptor antagonists and proton pump inhibitors)
				Decrease in testosterone may be beneficial in women; monitoring for hypogonadism is needed for men
				Careful review of other medications for potential drug-drug interactions is essential
				Monitor for AI
				Higher doses may be needed to counter escape
Levoketoconazole [4, 17]	BID	300–1200 mg/day (1200 mg/day)	US (FDA for CS)^b^	Risk of serious hepatotoxicity; monitor liver enzymes before and during treatment
				Risk of QTc prolongation
				Avoid use of gastric acid suppressors (e.g., H2-receptor antagonists and proton pump inhibitors)
				Decrease in testosterone may be beneficial in women; monitoring for hypogonadism is needed for men
				Careful review of other medications for potential drug-drug interactions is essential
				Monitor for AI
Metyrapone [4, 63]	TID or QID	500–6000 mg/day (6000 mg/day)	US (off-label use) EU (EMA for CS)	Rapid action
				Monitor for AI, hypokalemia, QTc prolongation, and hypertension
				11-deoxycortisol can cross-react in cortisol immunoassays
				Monitoring for hyperandrogenism is needed for women
Mitotane [4, 64]	TID	500–4000 mg/day (5000 mg/day in CD)	US (FDA for adrenal cancer with endogenous CS) EU (EMA for CS)	Slow onset of action and highly variable bioavailability
				Narrow therapeutic window
				11-deoxycortisol can cross-react in cortisol immunoassays
				Neurological toxicity is a limiting factor in some patients
				Potential limited use in women who could become pregnant, as teratogenicity and abortifacient activity, coupled with a long half-life have been observed
Osilodrostat [4, 6–10, 65]	BID	4–14 mg/day maintenance dosage (60 mg/day)	US (FDA for CD)^c^ EU (EMA for CS)	Rapid action
				Monitor for AI, hypokalemia, QTc prolongation, and hypertension
				11-deoxycortisol can cross-react in cortisol immunoassays
				Monitoring for hyperandrogenism is needed for women
*Intravenous treatment*				
Etomidate [4, 66]	–	0.04–0.1 mg/kg/h for patients in the ICU 0.025 mg/kg/h for patients not in the ICU	Off-label use only	Used for acute inpatient treatment of severe hypercortisolism
				Intravenous hydrocortisone is required at high doses to avoid AI

*AI* adrenal insufficiency, *BID* twice a day, *CD* Cushing’s disease, *CS* Cushing’s syndrome, *EMA* European Medicines Agency, *EU* European Union, *FDA* US Food and Drug Administration, *ICU* intensive care unit, *QID* 4 times a day, *TID* 3 times a day, *US* United States

^a^The approvals mentioned in this table are for US and EU, but many other countries have different drugs approved for CS

^b^In adult patients with CS for whom surgery is not an option or has not been curative

^c^In adult patients with CD for whom pituitary surgery is not an option or has not been curative

As with other helpful reviews that describe practical considerations for use of mifepristone [11], levoketoconazole [12], and pasireotide [13, 14], the objective of this narrative review is to address clinical considerations and recommendations [1] when using osilodrostat for patients with CD, ectopic CS, or adrenal CS by providing illustrative example case studies.

## Postsurgical management of recurrent CD

### Example case study 1

A 34-year-old female patient had typical clinical and clear biochemical evidence of CS, with elevations in both 24-h urinary free cortisol (UFC) and late-night salivary cortisol (LNSC) levels and a non-suppressed ACTH, confirming ACTH-dependent CS. A pituitary MRI revealed an 8-mm adenoma, supporting a diagnosis of CD. The patient underwent successful transsphenoidal surgery and was in remission for 2 years. Subsequently, she began gaining weight despite active weight management and developed insomnia and fatigue; LNSC levels were elevated at 1.8 × and 2.2 × upper limit of normal (ULN), and UFC was 2 × ULN, indicating clinical and biochemical recurrence of CD. Pituitary MRI revealed a newly visible 3-mm pituitary adenoma, but the patient did not wish to undergo repeat surgery; she was counselled about other treatment options and expressed interest in an oral treatment option that she would not need to bring to work during the day. Potassium and magnesium levels, as well as electrocardiogram (ECG), were normal, and she did not take other medications. The patient began osilodrostat treatment at 2 mg twice daily (BID), which was up-titrated to 4 mg BID in 6 weeks, and she achieved a normal UFC level within 3 months. She continued osilodrostat 4 mg BID treatment for 3 years and experienced decreased fatigue, improved sleep pattern, weight loss, and no adverse events (AEs) such as adrenal insufficiency (AI) or hypokalemia. However, after 3 years, ACTH levels tripled while UFC levels remained normal; serial MRI scans revealed that the pituitary tumor had grown to 6 mm. The patient subsequently agreed to surgery by an expert pituitary surgeon, discontinued osilodrostat, and underwent a successful second transsphenoidal surgery.

#### Clinical case points

##### Cortisol excess parameters to follow after surgical remission

In this example case study, both UFC and LNSC levels were abnormal when the patient was diagnosed and again were elevated with the recurrence 2 years later. However, UFC and LNSC elevations may not always be concordant in recurrent CD; in many patients, LNSC becomes abnormal before UFC, by up to 1 year in some studies [1, 15]. If UFC and LNSC are discordant, it is important to note which values were abnormal at initial diagnosis, as the same pattern is often, but not always, seen on recurrence and can be useful in treatment monitoring. For example, if UFC and LNSC are both elevated, confirming recurrence of CS, then continue to monitor both UFC and LNSC during treatment. If UFC is normal and only LNSC and/or cortisol following dexamethasone suppression are elevated, then in addition to clinical monitoring, serial monitoring of LNSC and/or cortisol following dexamethasone suppression is recommended to monitor for recurrence. UFC should also be periodically monitored, as it could become abnormal later in the disease course.

##### Baseline evaluation needed before medication start

This example patient provides a good illustration of the steps to be taken before initiating osilodrostat. Obtaining potassium and magnesium levels and ECG measurements are critical both before initiating treatment, as a baseline, and during treatment with osilodrostat; this is also true with other adrenal steroidogenesis inhibitors, such as metyrapone [10, 16] and levoketoconazole [12, 17] or with the glucocorticoid receptor blocker mifepristone [11], to monitor for the development of low potassium or prolonged QTc on ECG. For patients starting specifically on osilodrostat, an ECG should be performed at baseline and 1 week after initiating therapy to ensure there is no QTc prolongation (although this is uncommon, as described later), in addition to checking for hypokalemia or hypomagnesemia and, if present, correcting these before beginning treatment. An ECG should be repeated again after 2 to 4 weeks of treatment (depending on age, cardiac history, concomitant medications, and electrolyte abnormalities); if the ECG is normal, follow-up can be determined by the clinical situation, such as with dose increases. Potassium levels should be closely monitored with osilodrostat dose changes and checked more often if levels are out of the normal range or if other risk factors for hypokalemia are present. Abnormalities should be managed proactively—if levels are low, potassium replacement should be administered and levels followed to ensure that they normalize; alteration of the osilodrostat dose is not typically necessary. Patients with hypokalemia may also benefit from starting mineralocorticoid receptor antagonists (e.g., spironolactone or eplerenone). Of note, if osilodrostat treatment is interrupted, potassium and other concomitant medications should be closely monitored, as doses may need to be reduced or discontinued (see the “Treatment interruption for COVID-19” section).

##### ACTH monitoring during use of steroidogenesis inhibitors

This example patient’s tumor growth was noted when an MRI was performed because of a substantial increase in ACTH. Conducting regular follow-up to measure plasma ACTH levels has been recommended when using steroidogenesis inhibitors because of the dysregulation of the hypothalamic–pituitary–adrenal (HPA) feedback mechanism [1]. In our practice, periodic monitoring of ACTH levels at approximately 3- to 6-month intervals is used to assess for potential substantial increases. It is important to note that many factors impact ACTH values, which often fluctuate widely.

### Relevant published data—corticotroph adenoma considerations

When this example patient was started on osilodrostat, she had only a small residual tumor. It should be noted that adrenal steroidogenesis inhibitors do not directly target the pituitary ACTH-secreting adenomas, nor do they restore HPA axis circadian rhythm per se. On the basis of the known corticotroph growth that can occur in some patients following bilateral adrenalectomy, historically known as Nelson’s syndrome and more recently termed corticotroph tumor progression [2, 18, 19], there has been concern that medications blocking the production or action of cortisol may similarly trigger more aggressive growth of the adenoma. Because untreated corticotroph adenomas can gradually enlarge owing to natural history, it can be challenging to determine whether tumor growth on medical therapy is due to the treatment or would have occurred without the medication. A potential signal of tumor growth is progressive elevation in ACTH. However, the half-life of ACTH is short; as such, fluctuations in ACTH levels do not reliably correlate with tumor growth for patients on medical treatment [1]. For example, a study of 43 patients with CD receiving mifepristone daily demonstrated that elevated ACTH levels were common, but tumor progression was confirmed in only 4 patients [20]. However, promptly obtaining an MRI when abrupt, progressive ACTH increases are observed is advised.

Tumor size changes have been reported with osilodrostat. In vitro studies of osilodrostat showed no pituitary-directed effects on cell growth or ACTH production [21]. In LINC 3, the proportion of participants with ≥ 20% tumor volume increase (considered a significant change for clinical trials) was similar to the proportion with a decrease (33% vs 38%, respectively) in the 64 evaluable patients at week 48 [9]. Mean ACTH levels were higher than ULN at baseline and increased further during the study, but no association was noted between ACTH and cortisol concentrations at week 48 [9]. A case study also reported that a patient experienced increased tumor size 4 years after using osilodrostat despite no tumor size change until then [22].

Two retrospective studies of ketoconazole use have reported tumors becoming visible after treatment initiation [23, 24]: one retrospective study of 200 patients found that 8 (5%) had tumor size progression from undetectable to visible while on treatment [23]. In another retrospective study at a single center in France, medical therapy was stopped 12 to 30 months after treatment initiation in 5 patients without visible tumor at initial scan when the tumor became visible on therapy [24].

The phase 3 SONICS study evaluating efficacy and safety of levoketoconazole reported no study withdrawals due to tumor growth with no change in tumor size in most patients through 12 months on treatment [12, 25]. In this study, mean ACTH concentrations in the 80 patients with CD increased during dose titration and remained elevated through the maintenance phase. Importantly, there was no correlation between ACTH change from baseline to month 6 and UFC changes.

Pituitary tumor shrinkage has been observed with other CD medication therapies, such as pasireotide [26] and cabergoline [27], which target the corticotroph tumor, so these may be preferred in patients with large residual tumors who will be starting medical treatment.

Radiologic monitoring for corticotroph tumor progression is important in all patients treated with medical therapy, particularly as ACTH levels are impacted by many variables and may fluctuate. An MRI before treatment with adrenal steroidogenesis inhibitors (at baseline) followed by regular serial MRIs is advised to monitor pituitary tumor size in most patients. If no adenoma is observed, the timing of obtaining the next MRI depends on the complete clinical picture, but it is generally performed after 1 to 2 years, or sooner if ACTH levels increase substantially [1]. If an adenoma is present, repeating an MRI at 6 to 12 months after starting osilodrostat is advisable; if progressive tumor size is observed, osilodrostat treatment should be stopped and other management strategies suggested as done for this patient. A neurosurgical consultation is advisable with a multidisciplinary plan established based on patient preference, previous number of pituitary procedures, availability of expert neurosurgeons, and contraindications for surgery.

### Hypokalemia in the LINC trials

While the patient in this case example did not experience hypokalemia during several years of treatment, this electrolyte abnormality has occurred in clinical trials of osilodrostat treatment. In LINC 3, 18/137 participants (13%) experienced hypokalemia. Most serum potassium concentrations were maintained within the normal range, but a pattern was observed with greater decreases in mean serum potassium levels with increasing maximum concentration (C_max_) of osilodrostat [9]. Grades 3 to 4 hypokalemia were reported in 7 patients, with the lowest reported serum potassium concentration of 2.4 mmol/L (normal range was 3.5–5.3 mmol/L) [9]. Hypokalemia was treated with potassium supplements, spironolactone, dose reduction or interruption, or a combination of these approaches [9]. In our experience, if the potassium level is low-normal, then we may consider adding spironolactone, especially in women or in patients with hypertension. If the potassium level is below normal (i.e., < 3.5 mmol/L), then potassium supplementation should be administered, with follow-up lab tests to confirm normalization. Consideration can also be given to adding spironolactone to the regimen. While not common, osilodrostat dose interruption and intravenous potassium administration may be needed if more severe hypokalemia occurs and does not normalize with oral repletion and potassium-sparing medications.

## Choice of dose and titration speed

### Example case study 2

A 45-year-old male patient with a 1.2 cm intrasellar macroadenoma associated with clinical features of CD, 24-h UFC elevation of 2.4 × ULN, and LNSC 1.8 × ULN was in remission immediately after transsphenoidal surgery and had AI for 4 months; after the HPA axis recovered normal function, hydrocortisone was discontinued. Eight months after surgery, few symptoms of CS were present, but the patient again had high UFC (1.3 × ULN) and abnormal LNSC (1.5 × ULN). After assessing the patient’s current medication use, ECG results, and potassium and magnesium levels, the patient was started on osilodrostat 2 mg BID. Rapid up-titration to 4 mg BID within ~ 2 weeks was performed because there were no AI symptoms. The patient demonstrated signs of clinical improvement, such as 3-kg weight loss and improved sleep; however, UFC was still high (1.4 × ULN) and morning plasma cortisol was 15 µg/dL. Osilodrostat was subsequently increased to 6 mg BID, and the patient did well for 6 weeks. He then exhibited symptoms of an upper respiratory infection with fever (negative for coronavirus disease 2019 [COVID-19]) and developed fatigue, malaise, and severe headache, possibly suggestive of AI. He was not hypotensive, and serum chemistries remained normal. Morning plasma cortisol was 3 µg/dL as determined by a liquid chromatography tandem mass spectrometry (LCMS) assay, confirming the diagnosis of AI. Osilodrostat was stopped temporarily and the patient was given oral hydrocortisone 20 mg BID for 4 days. One week later, osilodrostat was resumed but at a lower dose of 5 mg BID. Upper respiratory infection symptoms resolved, and the patient did well.

#### Clinical case points

##### Gradual dose up-titration may decrease the risk of AI

This example patient developed AI when a rapid up-titration of osilodrostat coincided with an upper respiratory infection and fever. The effective adrenal blockade likely prevented the normal cortisol increase with the stress of illness and fever. This patient’s situation illustrates that unless the patient is seriously ill with CS/CD (not usually the case with a UFC that had recently increased from normal to 1.3 × ULN before osilodrostat treatment as his did), slow up-titration of osilodrostat after treatment initiation can be conducted. In this case, osilodrostat doses were increased quickly; however, in retrospect, as it was not urgent to gain control immediately, a slower titration of every 3 to 4 weeks or even longer would have been optimal to minimize the risk of AI. In our clinical practice, we typically start outpatients at a dose of 1 or 2 mg BID, depending on the clinical picture and baseline cortisol values; the dose is subsequently up-titrated based on both cortisol response and symptoms, usually at the next clinical visit in 2 to 8 weeks. Notably, some patients with very high UFC at baseline may experience a significant decrease in cortisol soon after initiation of the medication.

##### Adrenal insufficiency vs steroid withdrawal

Another challenge related to dose titration is differentiating glucocorticoid withdrawal vs AI, as both are conditions that can be observed along the same clinical continuum with overlapping symptoms (e.g., fatigue, lack of energy, nausea, loss of appetite, weight loss, myalgia, and abdominal pain) [28, 29]. Glucocorticoid withdrawal (i.e., symptoms associated with reduction in cortisol levels after pathological elevation for a prolonged period of time) is seen in many patients treated effectively for CS with either surgery or medication. Although the features of AI are similar to those of steroid withdrawal, AI is usually diagnosed on the basis of symptoms that may additionally include dizziness associated with lower blood pressure or postural vital signs, hypoglycemia, and very low morning cortisol values [28, 30–32]. Notably, symptoms of AI are usually, but not always, worse than steroid withdrawal symptoms. Hypotension and hypoglycemia can be particularly helpful for distinguishing these two conditions, as they are only observed in AI [32]. In the example case study, the patient was not hypotensive or hypoglycemic, but a low morning cortisol level confirmed AI [1]. When it is difficult to determine whether a patient has steroid withdrawal vs mild AI, down-titration of osilodrostat for a few days may improve symptoms and clinical status while follow-up biochemical evaluation is performed. Alternatively, if the symptoms are moderate to severe, interfering with the patients’ daily activities, discontinuation of osilodrostat and initiation of supplemental glucocorticoid replacement is advised. Supplemental glucocorticoids are always advised if suspicion for AI is high, while awaiting biochemical confirmation. As this case illustrates, monitoring patients for symptoms of AI during times of stress (i.e., fever, infection, physical stress/acute illness) is warranted because hypocortisolism can occur at any time during treatment with osilodrostat and particularly when there would normally have been HPA axis activation.

### Relevant published data—osilodrostat dose titration in randomized controlled trials

In the LINC 3 study, prescheduled dose up-titration steps within the first 3 months of treatment were mandated per study design, with a 2 mg BID starting dose of osilodrostat and up-titration occurring every 2 weeks until week 12 (with 5-, 10-, 20-, and 30-mg BID escalation) if 24-h mean UFC (mUFC) was greater than ULN. In contrast, in LINC 4, the starting dose of osilodrostat remained 2 mg BID, but it was up-titrated by central independent endocrinologists (used in this double-blind study) less rapidly at weeks 2, 5, and 8 on the basis of efficacy and tolerability [6, 9]. In LINC 3, for all patients, the median osilodrostat exposure was 130 weeks (range, 7–245 weeks) and median dose was 7.4 mg/d, which consistently decreased and normalized UFC [33]. In LINC 4, for all patients, the median osilodrostat exposure was 70.0 weeks (range, 2.0–112.7 weeks) and median dose was 5.0 mg/d, which maintained mUFC within normal range in most patients up to week 48 [6]. In LINC 4, increasing osilodrostat dose at 3-week intervals instead of every 2 (as in LINC 3) resulted in fewer hypocortisolism-related AEs (LINC 4 vs LINC 3: 27% vs 51%, respectively) without delaying the time to first mUFC normalization (LINC 4 vs LINC 3: 35 vs 41 days, respectively) [33, 34]. Notably, most cases (77%) of hypocortisolism-related AEs were reported to occur in the first 26 weeks during the titration or maintenance phase of the LINC 3 clinical trial, while 58% (7/12) occurred during the titration phase (the first 12 weeks) in LINC 4, supporting the use of a slower dose up-titration approach to avoid this side effect [33, 34]. Hypocortisolism-related AEs in LINC 3 and LINC 4 were predominantly of mild or moderate severity and were effectively managed via dose reduction, interruption, and/or concomitant glucocorticoid administration [34]. Only 3% of participants permanently discontinued osilodrostat because of these AEs in LINC 3 (4/137) and LINC 4 (2/73) [6, 9]. Together, these clinical data demonstrate that gradual up-titration of osilodrostat can mitigate the incidence of hypocortisolism-related AEs in many patients without affecting rates of UFC control. Alternatively, a lower starting dose of 1 mg BID may be used in patients with mild CS, which yields a slower dose titration as described subsequently in a later case.

### Monitoring for AI with osilodrostat treatment and the importance of assay method

Because osilodrostat is highly effective in rapidly achieving a prolonged blockade of cortisol secretion, it is important to carefully monitor for the risk of AI [35]. Evidence from 3 published cases demonstrated that delayed AI occurred even while patients were eucortisolemic on a stable dose of osilodrostat. The authors suggested that a rapid dose up-titration with a block-and-replace approach could also potentially minimize the AI risk [35] when it is considered important to gain control of cortisol overproduction carefully. There is some controversy about the use of this strategy, but it is reasonable to consider if the dose of osilodrostat will be escalated rapidly and to high doses. As seen with the example patient in Case 2, morning serum cortisol measurement is the preferred method utilized to confirm AI. It must be emphasized that UFC is not useful for this diagnosis because there is overlap in UFC levels between healthy normal people and those with AI at the low end of the normal range. Also, being aware of the type of assay used for cortisol measurement is very important; LCMS has a high specificity and can detect cortisol and cortisone and is preferred, whereas immunoassay has higher sensitivity but cannot differentiate them, potentially leading to “overestimation” of cortisol values [1]. Notably, in a patient taking osilodrostat or metyrapone, a normal morning cortisol level in an immunoassay lab test does not exclude AI, because of cross-reactivity with some adrenal precursors proximal to the 11β-hydroxylase enzyme blockade [1]. The assay used for the patient in this example was LCMS, so this was not a concern. His AI was confirmed with a low early morning cortisol level, and he responded well to treatment. As was done for this patient, prompt cessation of osilodrostat and treatment with glucocorticoids is needed in symptomatic patients while awaiting biochemical confirmation of AI to avoid life-threatening adrenal crisis if there is delay in obtaining cortisol results [36, 37].

## Increased testosterone with osilodrostat and drug-drug interactions

### Example case study 3

A 26-year-old female patient with CD had hirsutism at diagnosis. Because of persistent disease post-surgery, the patient initiated osilodrostat 2 mg BID. Six months after treatment initiation, the patient reported mild worsening of hirsutism. She did not want to take an additional medication for this and was comfortable using local hair removal methods. Testosterone levels were monitored and initially increased but subsequently returned to baseline one year after treatment initiation with no further intervention beyond local hair removal. Hirsutism gradually improved and was milder than at baseline. After a year she developed cluster headaches and was recommended to start verapamil; she remembered being advised to discuss all newly prescribed medications with her endocrinologist before therapy start. After the potential interactions between osilodrostat and verapamil were explained, including that her osilodrostat dose would likely be reduced, she decided to ask her neurologist for an alternative treatment for headache in order to not decrease the osilodrostat dose, which was controlling her CD very well.

#### Clinical case points

##### Pathophysiology and management of hirsutism in women on 11β-hydroxylase inhibitors

The osilodrostat-induced blockade of 11β-hydroxylase effectively inhibits adrenal cortisol biosynthesis and can increase testosterone by shunting the synthetic pathways proximal to the blockade toward androgen production [38]. The increased testosterone levels may induce or exacerbate hirsutism in some women. If the patient is seeking treatment for hirsutism, spironolactone can be added. Women who have persistent and bothersome hirsutism, even after addition of spironolactone, might benefit from switching to a treatment for hypercortisolemia with a different mechanism of action. In this case example, the patient had a mild increase in testosterone that resolved with time on continued treatment, did well with local removal of excess hair, and did not need additional measures.

##### Drug-drug interactions with osilodrostat

Although drug-drug interactions with osilodrostat are mild to moderate in many cases, it is important to be mindful of possible changes (both increases and decreases) in circulating levels of osilodrostat, depending on the effect of a concomitant medication on the metabolism of this drug. Likewise, osilodrostat may affect the metabolism of a newly prescribed medication (either increasing or decreasing it). It is essential to make the patient aware of the possibility of drug interactions and to report to the endocrinologist managing CS any new medications that have been prescribed or discontinued. Many frequently used concomitant medications to be aware of when treating patients with osilodrostat are summarized in Table 2.

**Table 2:** Concomitant medications with potential to interact with osilodrostat [2]

Interaction with	Medication		Rationale
CYP3A4 substrates	Atorvastatin^a^ Aprepitant^a^ Buspirone^a^ Conivaptan^a^ Cyclosporine Eletriptan^a^ Eplerenone^a^ Ergot alkaloids Erythromycin Fentanyl Glucocorticoids Ibrutinib^a^ Midazolam^a^ Nifedipine Omeprazole	Ondansetron Propranolol Quetiapine^a^ Quinidine Ritonavir Sildenafil^a^ Sirolimus^a^ Testosterone Ticagrelor^a^ Verapamil Vincristine Voriconazole Warfarin Zolpidem	Osilodrostat is a weak CYP3A4 inhibitor and may increase plasma concentrations of CYP3A4 substrates
CYP3A4 inhibitors	Amiodarone Aprepitant Atomoxetine Cimetidine Clarithromycin^b^ Conivaptan Diltiazem Erythromycin Grapefruit juice^b^	Itraconazole^b^ Ketoconazole^b^ Metyrapone Nefazodone^b^ Omeprazole Ritonavir^b^ Verapamil Voriconazole^b^	Osilodrostat dose should be increased when discontinuing a strong CYP3A4 inhibitor
			Osilodrostat dose should decrease by 50% when co-administering with a strong CYP3A4 inhibitor
CYP3A4 inducers	Carbamazepine^c^ Dexamethasone^c^ Enzalutamide Mitotane^c^ Nevirapine Oxcarbazepine	Phenobarbital^c^ Phenytoin^c^ Pioglitazone^c^ Rifampin^c^ St. John’s wort	Osilodrostat dose may need to be increased when co-administered with a strong CYP3A4 inducer
			Osilodrostat dose may need to be decreased when discontinuing a strong CYP3A4 inducer
CYP2B6 inducers	Carbamazepine^c^ Insulin Modafinil Nafcillin	Omeprazole Rifampin Tobacco	Osilodrostat dose may need to be increased when co-administered with a strong CYP2B6 inducer
			Osilodrostat dose may need to be decreased when discontinuing a strong CYP2B6 inducer
CYP1A2 and CYP2C19 substrates	CYP1A2 Acetaminophen Amitriptyline Caffeine^a^ Clozapine Duloxetine^a^ Estradiol Haloperidol Imipramine Melatonin^a^ Naproxen Ondansetron Propranolol Ramelteon^a^ Theophylline Tizanidine^a^ Verapamil Warfarin Zolmitriptan	CYP2C19 Amitriptyline Citalopram Clobazam Clopidogrel Diazepam Indomethacin Labetalol Omeprazole^a^ Phenytoin^a^ Progesterone Propranolol Warfarin	Osilodrostat may increase plasma concentrations of drugs metabolized by CYP1A2 and CYP2C19
CYP2D6 and CYP2E1 substrates	CYP2D6 Amitriptyline Atomoxetine^a^ Carvedilol Chlorpromazine Citalopram Dextromethorphan^a^ Donepezil Haloperidol Imipramine Lidocaine Metoclopramide Oxycodone Propafenone Propranolol Tolterodine^a^ Venlafaxine^a^	CYP2E1 Acetaminophen Ethanol Halothane Isoflurane Theophylline	Osilodrostat may increase plasma concentrations of drugs metabolized by CYP2D6 and CYP2E1

^a^Sensitive substrate

^b^Strong inhibitor

^c^Strong inducer

This case example illustrates the value of patient education, as the example patient in this case study remembered to contact her endocrinologist when a new medication was prescribed for headache. After learning about the potential interactions, she was able to make an informed decision to choose another medication. It can be helpful to remind patients about this at each visit.

### Relevant published data—hirsutism with adrenal steroidogenesis inhibitors

Hirsutism may occur with increased testosterone levels and is a known potential side effect of metyrapone and osilodrostat treatment [8, 39, 40]. However, as seen in clinical practice and in the patients in the LINC studies, preexisting hirsutism remains stable or improves in the large majority of patients after starting therapy with osilodrostat [41], possibly resulting from reduced production of other androgens such as 11-oxyandrogens [42]. In LINC 2, an increase in testosterone was observed in 9 of the 12 female patients who completed 22 weeks of treatment; 5 female patients reported new/worsening hirsutism and/or acne with testosterone levels > ULN at week 22 [8]. Long-term data from LINC 2 showed that mean testosterone levels decreased to baseline levels at last assessment (up to month 70 of the extension phase) despite continued treatment [43]. In the larger LINC 3 study, hirsutism improvements were observed among female patients taking osilodrostat regardless of mUFC control, occurring in 27% of females at week 24 and 34% of females at week 48. Testosterone levels initially increased followed by a return to within the normal range during the extension period (weeks 48–72), with a mean (SD) testosterone level of 0.8 (0.7) × ULN at week 72, which was similar to baseline testosterone values in women; 12 female patients reported an AE of mild hirsutism, with none leading to discontinuation [33]. That was the experience of the patient in this case study; there was an increase in testosterone and hirsutism, but it was managed with local treatment and gradually improved both biochemically and clinically.

In male patients, hypogonadism from CS may be present before starting medical treatment. Osilodrostat was associated with testosterone rising to near the normal range in LINC 2 and LINC 3, potentially because the hypothalamic-pituitary–gonadal axis dysfunction improved as cortisol decreased with treatment; no apparent changes were observed further in the extension phase [8, 33].

### Relevant published data—drug-drug interactions with osilodrostat

A key concept when considering which agent to use in the medical management of CS is to review the patient’s other medications for potential drug-drug interactions that may cause clinical issues. This is true when initiating a new medication for CS, as well as when a patient taking a medical therapy for this disorder starts a new medication from another health care provider. Osilodrostat is a weak cytochrome P450 3A4 (CYP3A4) inhibitor. For use with strong CYP3A4 inhibitors (e.g., clarithromycin, which is also a CYP2B6 inducer), it is recommended to use a low osilodrostat starting dose (or down-titrate dose if a strong CYP3A4 inhibitor is added while on therapy) [2, 40]. When prescribed with medications that are CYP3A4 substrates (e.g., atorvastatin, verapamil [as in the case example patient]), osilodrostat should be used with caution as it may increase plasma concentrations of those medications, thereby increasing the risk of side effects [2]. For concomitant use with medications that are CYP1A2 substrates (e.g., amitriptyline), caution and a slow-titration approach is recommended [2, 40]. Medications that are CYP2D6 substrates (e.g., citalopram) also require close monitoring, as osilodrostat may increase plasma concentrations of drugs metabolized by CYP2D6 and CYP2E1 [2]; monitoring for side effects and consideration of dose reduction in discussion with the prescribing physician are advised. Prolonged QTc interval may be observed with osilodrostat treatment. Therefore, when the patient is taking concomitant medications that are known to prolong the QTc interval (e.g., some antiarrhythmics, fluoroquinolones, macrolides, some antidepressants), more frequent ECG monitoring may be needed [2]. Monitoring electrolytes is essential to prevent QTc prolongation. In the LINC trials, few instances of QTc prolongation were reported [8, 9], with 5 patients (4%) having elevated QTcF values but < 480 ms at all time points [9].

## Treatment interruption for COVID-19

### Example case study 4

A 45-year-old male patient with CD was well controlled clinically and biochemically on osilodrostat 2 mg BID. Subsequently, the patient was diagnosed with COVID-19. Initially, he had minimal symptoms and osilodrostat was stopped; no glucocorticoids were provided, as there were no signs of AI. Two days later, severe COVID-19 symptoms and hypoxia developed, and he was given high-dose glucocorticoids as treatment for COVID-19. The patient was off osilodrostat for 6 weeks after recovery from COVID-19 and then restarted on the original dose. He was given oral glucocorticoids and a home glucocorticoid injection kit for use during a future intercurrent infection or other events during which stress dosing may be needed.

#### Clinical case points

##### Patient education about AI risk and management during illness

All patients on adrenal steroidogenesis inhibitors should have a prescription for hydrocortisone tablets (or another glucocorticoid preparation) and a stress-dose injectable kit at home in case AI develops. For patients with severe symptoms of AI, higher doses of hydrocortisone are needed (2–5 × usual replacement doses). If persistent severe diarrhea or vomiting occur, patients without immediate access to an emergency room should inject glucocorticoids and then be seen in an urgent care or emergency department as soon as possible. As part of patient education, patients should be prepared to explain in this setting that they have CS/CD and are taking a medication that blocks cortisol production so need to be given glucocorticoids.

For patients with CS and acquired COVID-19, it is recommended that a patient be given stress doses of glucocorticoids [44]. Although there are no data specifically addressing this point, stopping osilodrostat treatment is likely advisable; the optimal duration of treatment interruption during COVID-19 or other severe infections has not been established. Patients with COVID-19 have highly variable presentations and recoveries; data on long COVID-19 in the setting of CS are needed but are lacking at this time. Thus, an individualized approach to restart concomitant medications and reinstitute glucocorticoid-lowering drugs once the patient is stable clinically seems advisable.

All patients should be monitored for drug-drug interactions with medications used to treat COVID-19. Tests advised include an ECG and measurements of cortisol, electrolyte, and magnesium levels. If osilodrostat therapy needs to be interrupted, it is important to monitor and potentially adjust concomitant patient medications. For example, for patients on potassium, spironolactone, and insulin/antidiabetic therapies, stopping osilodrostat treatment may require lowering the potassium and spironolactone dose and increasing insulin or other antidiabetic therapy. Similarly, close monitoring and concomitant medication dose adjustment is needed when osilodrostat is restarted and with each dose up-titration.

### Relevant published data—treating COVID-19 in patients with CS

Given the potential risk of AI with medical therapy for CS, situations that can potentially precipitate adrenal crisis, such as infection, trauma, surgery, or significant psychological distress, warrant consideration of temporarily halting any CS treatment, including osilodrostat [32, 37]. Individuals with AI have depleted innate immunity, which can increase risk of infections such as COVID-19 and worsen COVID-19 symptoms [45–47]. Additionally, mild COVID-19 symptoms are similar to those of AI, which could lead to unnecessary utilization of glucocorticoids [45]. For this reason, considering nuances related to the correct timing of stress-dose glucocorticoid administration to achieve the intended therapeutic effect is important for patient outcome [45]. Of note, there may be independent effects of COVID-19 on the HPA axis, even in patients without CS. Biopsies performed on the adrenal glands of patients who had died from COVID-19 showed that the viral infection targets the adrenal gland and can lead to cellular damage, potentially resulting in adrenal dysfunction that is sometimes seen during the acute phase of COVID-19 [48]. After recovery from COVID-19, resumption of medical therapy for preexisting comorbidities and use of cortisol-lowering drugs are advised for favorable long-term patient outcomes [49].

### Treating CS during the COVID-19 pandemic

During the height of the COVID-19 pandemic surges, imaging and laboratory testing for the diagnosis of CS in patients with mild clinical features was sometimes deferred [44], leading to delays in diagnosis. Once the diagnosis of CD is confirmed, if pituitary surgery cannot be done because of local COVID-19 pandemic-related restrictions, medical therapy is advisable for patients with active CD.

Although robust clinical evidence is not yet available, published case studies demonstrate clinical challenges in managing patients with CS and COVID-19. For example, antiviral drugs and antibiotics used to treat COVID-19 can interact with drugs used for CS. A 67-year-old male with CD and acquired COVID-19 was maintained on 0.5 mg cabergoline and 500 mg metyrapone daily for CD; he was also treated with azithromycin, ceftriaxone, and hydroxychloroquine for COVID-19 [50]. During hospitalization, symptomatic hypotension with hypoglycemia indicated possible AI. Cessation of metyrapone treatment and providing 50 mg of intravenous hydrocortisone stabilized the patient [50]. Notably, other potential drug-drug interaction effects include QTc prolongation (e.g., the antiviral drug ritonavir and CS drugs pasireotide, ketoconazole, and levoketoconazole can prolong the QTc interval), liver toxicity (e.g., ketoconazole is metabolized by CYP3A4 cytochrome, and the antibiotic azithromycin may increase ketoconazole concentrations), and hypokalemia (in combination with pasireotide, hydroxychloroquine, and chloroquine) [25, 50, 51]. No contraindications with COVID-19 vaccines have been reported with osilodrostat [40]. As previously mentioned, ECG should be performed at treatment initiation and serially thereafter as needed; this is particularly important with concomitant medications that may prolong the QTc interval, especially in patients with electrolyte abnormalities.

It is also important to optimize medical treatment for preexisting comorbidities and to choose drugs with the potential to have a positive effect on glucose, blood pressure, and/or weight. Improving not only the hypercortisolemia, but also focusing on optimal control of comorbid conditions, may decrease the complications associated with COVID-19 [49]. Given the limitations on face-to-face visits during COVID-19 restrictions, virtual health care provider visits can be beneficial to triage patient needs [51]. Laboratory tests can be temporarily deferred on the basis of clinical assessment and/or performed at local centers [51]. Virtual follow-ups have been recommended for interim medical monitoring [51] and are welcomed by many patients who may live far from major referral centers; thus, it would be beneficial if they can continue even beyond the pandemic.

## Timing of medication administration

### Example case study 5

A 34-year-old female patient with mild CD underwent transsphenoidal surgery. Immediately postoperatively, cortisol was 16 µg/dL with no glucocorticoid replacement, indicating lack of remission. At 2 months after surgery, UFC was still elevated 1.3 × ULN, and LNSC levels were 1.5 × and 1.7 × ULN. Because of persistence of mild hypercortisolemia, she was started on osilodrostat 1 mg BID. The patient then experienced clinical improvement with normal UFC levels; however, she had persistent insomnia. Osilodrostat dosage was increased to 2 mg at night while continuing 1 mg in the morning, resulting in improved sleep pattern, as well as biochemical control (LNSC and UFC normalization).

#### Clinical case points

##### Dosing considerations—lower starting dose for patients with mild CS; higher evening dose in some patients

Although the US Food and Drug Administration (FDA)–approved starting dose of osilodrostat is 2 mg BID, clinical experience thus far has shown that some patients do well when initiated on a lower dose. The patient in this case is an example of someone who attained biochemical control on a low dose of medication. Some patients with recurrence or early/mild disease present with elevated LNSC level, yet have normal or minimally elevated UFC level, so both tests can be helpful in disease management. Starting at 1 mg BID in patients with mild to moderate cortisol elevations may allow some patients to reach biochemical control at lower doses while reducing the risk of AI. Another interesting approach that has been tried, as seen in this case example, is to give a higher dose of osilodrostat in the evening when the patient either has borderline or intermittent hypercortisolemia or persistent symptoms. The concept is that it might improve control of hypercortisolemia and help restore normal circadian rhythm in some patients [1]. Data are needed to confirm both of these clinical approaches.

### Relevant published data—LNSC for diagnosing and monitoring treatment of CD and circadian rhythm considerations

For healthy individuals with a normal day/night cycle, the HPA axis circadian rhythm has a peak in cortisol early in the morning and a nadir between 11:00 PM and 1:00 AM; because an early sign of CS is lack of this late-night circadian nadir, monitoring LNSC can be beneficial in addition to monitoring UFC values [52, 53].

It is not clear whether targeting diurnal secretion assessed by morning cortisol or LNSC is meaningful because there is limited evidence demonstrating that treatment administered at night facilitates restoration of HPA axis circadian rhythm [1]. Nonetheless, some experts use this approach in select patients, with published evidence indicating that normalization of both LNSC and UFC may be a target of management with medical therapy. For example, an exploratory analysis from a phase 3 trial of patients with CD treated with pasireotide long-acting release showed that patients who had both mean LNSC and mUFC controlled at month 12 had greater improvements in systolic/diastolic blood pressure and weight reduction than those with uncontrolled mean LNSC and mUFC [54]. In another exploratory analysis of a different phase 3 trial of patients with CD treated with subcutaneous daily pasireotide, LNSC levels decreased rapidly, with a median decrease of 47% by month 3 in patients with elevated LNSC at baseline; LNSC levels normalized in 36% of patients (24/67) after 6 months and 39% of patients (13/33) after 12 months [55]. Further, in patients with adrenal incidentalomas and mild autonomous cortisol secretion who were given metyrapone specifically in the evening, the cortisol rhythm was “reset,” with reduced cortisol levels at night and normalized levels comparable to those of healthy controls [56]. In the case example, the patient’s insomnia was improved with a higher nocturnal dose of osilodrostat; further investigation about this approach is needed.

## Ectopic CS

### Example case study 6

A 68-year-old male patient with history of progressive severe weakness, edema, shortness of breath, hypokalemia of 2.9 mEq/L, and weight loss of 40 lb over several months had UFC 15 × ULN and ACTH 10 × ULN, confirming the presence of ACTH-dependent CS. His cortisol did not suppress with high-dose dexamethasone. The patient underwent pituitary MRI, computerized tomography (CT), positron emission tomography CT (PET-CT), and Ga-68 DOTATATE PET/CT, but no source for ACTH excess was found. Owing to clinical deterioration and comorbidities (e.g., heart failure, severe hypertension), the patient was not a candidate for urgent bilateral adrenalectomy. He was started on osilodrostat 10 mg BID (high starting dose) and hydrocortisone 20 mg BID (block-and-replace approach) to allow for higher dosage with faster blockage of adrenal steroidogenesis.

#### Clinical case points

##### Considerations in severe CS

This example patient has severe, occult ectopic ACTH excess as the source of his CS and, given the severity of hypercortisolemia, it is important to seek rapid control medically in such cases. The hypokalemia experienced by this example patient is common in similar cases and can be life-threatening. Proactive management of potassium levels is essential in all patients, but in severe CS, especially ectopic CS, frequent monitoring for hypokalemia is essential. Low potassium should be corrected aggressively before the initiation of osilodrostat and during therapy as needed. Use of osilodrostat in a block-and-replace approach for patients with severe CS (patients experiencing severe symptoms and in need of immediate cortisol normalization) can facilitate rapid biochemical control while also preventing the development of AI.

### Relevant published data—adrenal steroidogenesis inhibitors in ectopic and adrenal CS

The FDA approved osilodrostat for treatment of CD when pituitary surgery is not an option or has not been curative. Use of osilodrostat for urgent control of ectopic CS has been reported in published observational and case studies outside of the United States. In Japan, in a phase 2, single-arm, open-label study of 9 patients with endogenous CS not caused by CD (5 patients with adrenal adenoma), osilodrostat reduced mUFC at week 12 and was generally well tolerated [57]. In a case series of 3 patients with severe non-pituitary CS in France, osilodrostat rapidly and safely controlled severe hypercortisolism with a block-and-replace approach using a high dose of ≥ 6 mg/day; this dose was suggested for patients with cancer, ectopic CS, and baseline cortisol > 36 µg/dL (normal: 7–18 µg/dL) [58]. Seven patients with CS due to cortisol-secreting adrenocortical carcinoma were also treated with osilodrostat in another case series in France [59]. At daily doses of 4 to 40 mg, treatment was well tolerated and fully controlled hypercortisolism in all 7 patients, occurring between 1 week and 3 months [59]. High-dose osilodrostat as a first-line monotherapy was also used in a patient admitted to an intensive care unit in France with a life-threatening presentation of ectopic CS [60]. Treatment initiated with 20 mg/day was followed by 5-day block-and-replace approach with maximum dose of 60 mg/day; cortisol levels normalized in 6 days, ultimately allowing for rapid discharge from the intensive care unit [60].

Triple therapy with other adrenal steroidogenesis inhibitors has also been used in severe CS. In a cohort study of 11 patients with ACTH-dependent CS and severe hypercortisolism, concomitant mitotane, metyrapone, and ketoconazole was an effective alternative to emergency bilateral adrenalectomy [61]. Of note, a 39-year-old female patient with a neuroendocrine tumor complicated by diabetic ketoacidosis, pneumonia, and herpes zoster was maintained on this therapy for 1 month. The patient then underwent exploratory surgery, which led to remission of hypercortisolism [61].

## Conclusions

Many factors, including rapidity of response needed, concomitant medications, tolerability, ease of use, probability of biochemical normalization, demonstrated clinical improvement, local availability, approval, and cost, should be considered when initiating any therapy for CS. When osilodrostat, a potent adrenal steroidogenesis inhibitor, is used, frequent assessment for potential AI and side effects due to increase in adrenal precursors, which may lead to hirsutism, edema, hypertension, or hypokalemia, should be conducted. If these occur, potassium replacement, downward adjustment of osilodrostat, interruption of treatment, and/or temporary addition of glucocorticoids can be effective. Educating patients about the symptoms and treatment of AI, the possibility of drug-drug interactions, and when to call their health care provider can enhance patient safety. Osilodrostat provides both biochemical and clinical improvement in many patients with CS/CD. Future research and clinical experience are needed to expand current knowledge about this medication.
